# Bandgap engineering and high-throughput defect dynamics in two-terminal CsGeI_3_-Si monolithic tandem solar cells

**DOI:** 10.1038/s41598-026-56282-2

**Published:** 2026-06-18

**Authors:** Sujata Singh, Gun Anit Kaur, Mamta Shandilya, Ajit Sharma, Pravin Kumar Singh

**Affiliations:** 1 School of Physical Sciences, Indian Institute of Technology Mandi, Mandi, Himachal Pradesh 175075 India; 2 School of Physics & Materials Science, Shoolini University, Solan, Himachal Pradesh 173229 India; 3 Department of Chemistry, School of Chemical Engineering and Physical Science, Lovely Professional University, Jalandhar, Punjab 144411 India; 4 Department of Physics, Manipal University Jaipur, Jaipur, Rajasthan 303007 India

**Keywords:** Perovskite, Tandem solar cell, Monolithic, Band gap, Efficiency, Energy science and technology, Engineering, Materials science

## Abstract

Simulation-driven investigations are presented on highly efficient monolithic tandem solar cells with climate-efficient nano-scaled perovskite and crystalline silicon for green energy generation. Tandem solar cells comprise a lead-free CsGeI_3_ perovskite top cell and a silicon bottom sub-cell. A Cu_2_O hole transport material layer and a ZnO electron transport material layer was used. Optimizations were performed by varying doping, defect concentration, thickness, and band gap to obtain valuable insights into material properties. These perovskite-silicon tandem solar cells, with a wide band gap and optimized parameters, yielded power conversion efficiencies above the Shockley-Queisser limit for single-junction cells. The structure of perovskite-silicon tandem solar cells is Glass/FTO/ZnO/CsGeI_3_/Cu_2_O/RL/Si(p^+^)/Si(p)/Si(n)/Au. After optimization, the results show a power conversion efficiency of 37.23%, a J_sc_ of 23.95 mA/cm^2^, a Voc of 1.895 V, and an FF of 82%. This research shows that through this hybrid perovskite-silicon technology, one is assured of increased energy output while decreasing carbon footprints and increasing renewable energy use.

## Introduction

Annual carbon dioxide emissions from energy and industry are 87%, so a low-carbon energy system is imperative for combating climate change. Clean energy is important not only for health but also for global development. The population is without access to power constitutes 10%, and 41% is without access to clean cooking fuels. Outdoor air pollution results in deaths estimated at 4 to 10 million annually^1^. Global energy consumption keeps increasing as the International Energy Agency predicted in 2021. According to data, in 2022, the figure of global energy users crossed 8 billion and by 2050 it will be nearly 9.7 billion^2^. Global climate change reports warn of the urgent need to protect the Earth^3^. Transitioning to green renewable energy, which is both limitless and sustainable, is now essential because non-renewable energy sources (crude oil and natural gas) will eventually deplete. Photovoltaic solar cells are becoming an increasingly popular choice among green renewable energy technologies and offer several benefits, including being renewable, cost-effective, and environmentally friendly^4^. Research in this field is attracting an increasing number of researchers seeking to address climate issues and contribute to the development of a green future for energy^5^. According to the U.S. Department of Energy’s Solar Energy Technologies Office (SETO), the amount of electricity produced by solar cells is expected to increase to 1600 gigawatts by 2050^6^, and recent research has shown a marked increase in the efficiency of perovskite solar cells. They are attractive for use in green renewable energy due to their low manufacturing costs, good performance, large light absorption coefficients, long carrier diffusion lengths, and a large bandgap; as a result, they hold the potential to exceed the 31% efficiency limit set by the Shockley-Queisser Theory^7–9^. However, perovskite solar cells have limitations related to their stability, hysteresis effects, and structural design^10^. Although lead-based perovskite solar cells have received a lot of attention due to their high efficiencies and low manufacturing costs, the toxicity and hazards associated with lead present significant health and environmental risks^11^. Tandem Solar Cell (TSC) was first reported in 2006 and has since proven very useful for addressing the limitations of limited light absorption and thermal losses in solar cells^12^. The efficiency of these cells exceeds 45%, according to the Shockley-Queisser Limit Theory^13^. TSCs are constructed using two subcells made of different absorbers stacked together. They capture a broader spectrum of sunlight than single solar cells^14,15^. A typical efficient tandem device includes a wide bandgap (1.5–2.0 eV) perovskite top cell, and a bottom cell with a small bandgap (1.15–1.35 eV) and a strong solar light absorption spectrum^14,15^. This type of structural design improves solar light absorption efficiency. There is currently a variety of designs for TSC, and further improvements of their efficiency could be achieved by selecting low-reflectivity, tunable-bandgap materials and high-quality hole transport layers (HTLs), electron transport layers (ETLs), and other cell-layer materials^16–18^. For monolithically integrated tandem cells, which do not have gaps between the cell layers, current matching between multiple junctions is necessary to allow for uniform flow of current through all junction layers. The efficiency of photovoltaic devices depends on many factors, including, but not limited to, doping concentration, the amount of current generated by free carriers through diffusion and drift, recombination processes^19^, diffusion length, polycrystalline grain size and grain boundaries, and the surface area and thickness of the cell^20,21^. The selection of materials for ETL and HTL requires a thorough analysis of parameters such as electron affinity, band gap, doping concentration, absorption coefficient, defect density, and exciton diffusion length. This study used Zinc Oxide (ZnO) as the ETL and Copper (I) Oxide (Cu_2_O) and Cesium Germanium Triiodide (CsGeI_3_) to create the HTL. ZnO and Cu_2_O/CsGeI_3_ were selected for their high transparencies, allowing light to reach the surface of the active layer completely and enabling control of their properties.

It is well recognized that hole and electron transport in inorganic semiconductors is less efficient compared to crystalline semiconductors. Consequently, carrier mobility in crystalline semiconductors ranges between 15 and 0.1 cm^2^/V s. The bottom cell of the solar device in this work is a crystalline silicon cell with varied doping concentrations aimed at enhancing performance. Silicon has been widely recognized as one of the key photovoltaic materials because it offers stability, high efficiency, and abundance. In the case of tandem devices, silicon can absorb the light reflected from the semi-transparent top layer; therefore, it is appropriate for use in the bottom cell. The promising nature of crystalline silicon is the enabling basis for its application, but the flexibility of perovskite-silicon-based solar cells is also under evaluation today^22^. A critical review of the available literature led to an improved understanding of these devices in this paper^23^. This study simulated a two-terminal TSC with a CsGeI_3_-perovskite/crystalline silicon (c-Si) active layer using SCAPS 1D software. The value of this device model is that it allows for tuning of the band gap and optical properties of the perovskite and silicon cells. Using a device structure model with a 1.6 eV band gap perovskite layer, we estimated a conversion efficiency of about 37%. This study also demonstrates the design of high-efficiency nano-optical structures and reflective coating techniques for perovskite-silicon tandem solar cells^24^.

## Device modeling and simulation methodology

**Fig. 1:**
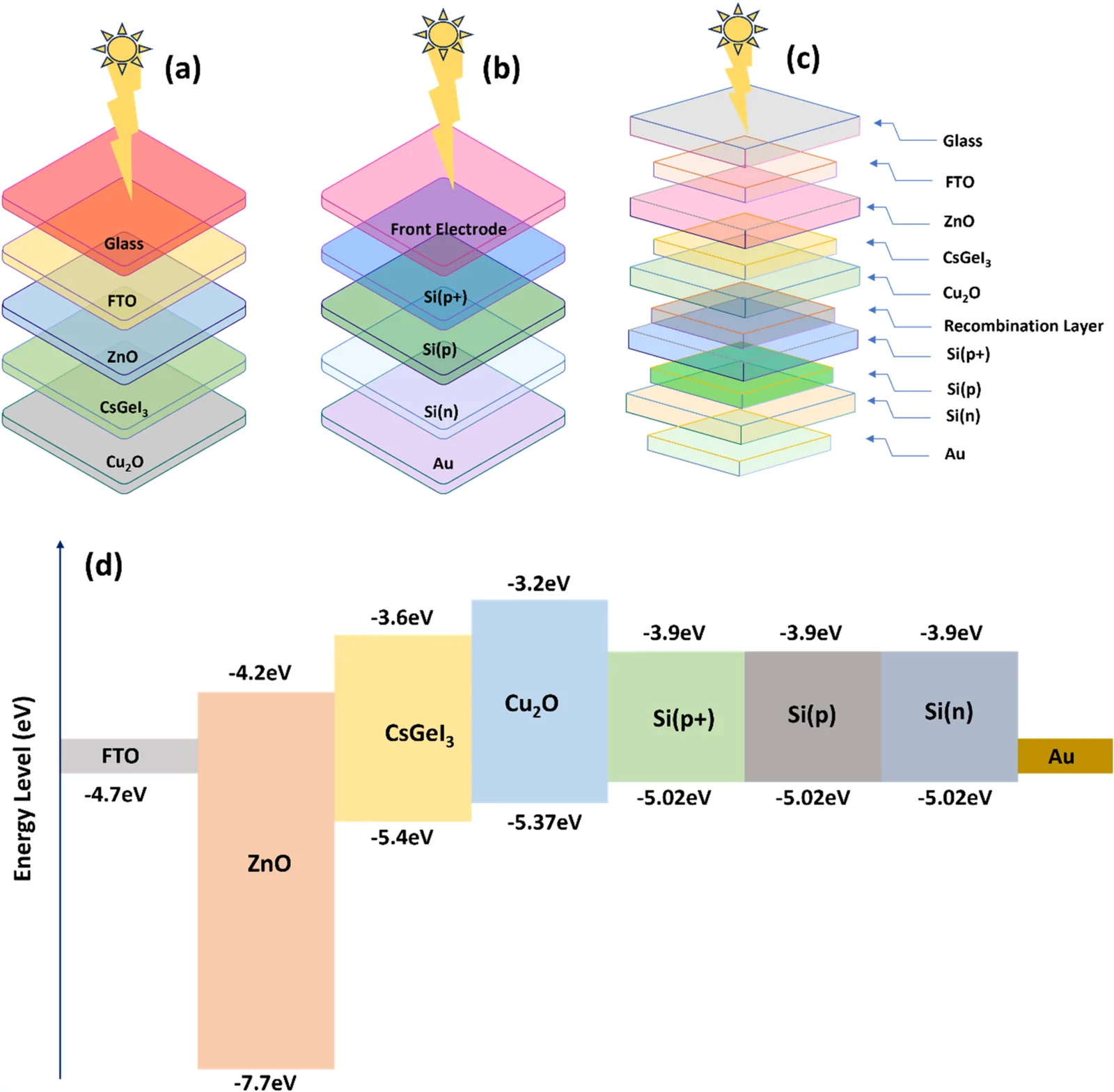
Schematic illustrations and energy band alignment in Glass/FTO/ZnO/CsGeI_3_/Cu_2_O/RL/Si(p^+^)/Si(p)/Si(n)/Au tandem solar cells: (**a**) *Single Junction Top Cell*: Illustration of a CGI perovskite solar cell equipped with Hole Transport Layer (HTL) and Electron Transport Layer (ETL). (**b**) *Single Junction Bottom Cell*: Depiction of a silicon solar cell featuring various doping levels. (**c**) *Monolithic Tandem Solar Cell Structure*: A detailed representation of a tandem solar cell composed of nine layers by integrating the top and bottom cells. The yellow arrow symbolizes the influx of sunlight. (**d**) *Energy Band Alignment Chart*: Graphical representation of the energy band alignments of all materials utilized in the solar device.

Solar Cell Capacitance Simulator (SCAPS) software offers several advantages, including heterostructure framing across multiple layers, a user-friendly interface and control, and the ability to operate in both dark and light environments. This simulation is executed using SCAPS-1D version 3.3.10, under A.M 1.5 G light spectrum, prepared by Gent University, Belgium^25,26^. Simulations were equilibrated under conditions approximating standard test conditions for solar cells, using a temperature of 300 K (near the STC reference of 25 °C), an AC perturbation frequency of 1 MHz, and a DC forward bias of 1.5 V. Temperature-dependent simulations were additionally performed, as detailed below, to assess thermal sensitivity. In this simulation, optical reflection losses at the interfaces and parasitic resistances were neglected in order to focus on the intrinsic material performance and carrier transport behavior of the tandem solar cell. In practical devices, interface reflections and parasitic resistances may introduce additional losses, which could slightly reduce the overall device efficiency^55^. In the simulations, ohmic contacts were assumed at both electrodes to facilitate efficient carrier extraction. Carrier transport across the interfaces was modeled using the thermionic emission mechanism implemented in SCAPS. Numerical convergence of the simulations was achieved using the default SCAPS solver settings with standard tolerance limits. The absorber layer plays a significant role in providing essential information on the open-circuit voltage (V_oc_), short-circuit current (J_sc_), and the diffusion length of electron-hole pairs. Three separate simulations were performed on different devices: the top, bottom, and tandem solar cells (TSC). These simulations were conducted under conventional solar illumination conditions of A.M. 1.5 G, with no applied voltage (Fig. 1).

Figure 1a displays the configuration of a single-junction CGI perovskite solar cell. It includes a glass substrate, a fluorine-doped tin oxide (FTO) layer, a 70 nm thick ETL composed of ZnO, a central 0.9 µm thick perovskite CsGeI_3_ absorber layer, and a 0.3 µm thick Cu_2_O HTL^27^. This can exhibit excellent compatibility and optical properties with HTL (deposited on top) and ETL (coated underneath), enabling integration with the perovskite layer^28,29^. Cu_2_O outperforms NiO and PTAA as HTL due to its small valence band offset with CsGeI_3_ for better hole extraction, backed by UPS data. ZnO ETL gives a conduction band offset (CBO = 0.99 eV) ideal for electron collection^30,31^. Cu_2_O enables low-cost SILAR deposition with type-II band offsets (VBO ≈ − 0.43 eV; CBO ≈ − 0.99 eV; DFT: Cu_2_O VBM ~ 5.4 eV, CsGeI_3_ ~5.0–5.6 eV), matching planar perovskite devices reaching 8.23% efficiency. The mild conduction band cliff is controlled via interface defect densities, while Cu_2_O’ s high hole mobility and thermal stability versus organic HTLs support fully inorganic tandem fabrication^32^. The materials were chosen by reviewing relevant literature and carefully analyzing the results through multiple layers of simulation. Figure 1b, the lower device is shown with a 1.8 μm P+ (heavily doped silicon) back surface field (BSF) layer, a 0.9 μm p-type-doped absorber of the standard p-bulk type, and a very thin n-type conductivity-shaped emitter (0.04 μm thick); simulations have optimized both thicknesses to enhance the performance of solar cells.

Figure 1c presents a more compact layout of the monolithic (2T) TSC layer configuration. The critical requirements for device assembly are described in depth in Table 1, while Table 2 contains the key defects. The p-i-n top cell and the p(+)-p-n bottom cell layout define the device. The band alignment in the band diagram of the device is also essential for providing insight to the HOMO at the hole acceptor material^33,34^, which plays a vital role in V_OC_. In terms of efficient photoelectron extraction, it is required that the electron affinity of the perovskite absorber layer be greater than the electron affinity of the ETL but less than the electron affinity of Cu_2_O^33,34^, as can be seen in the band diagrams of ZnO/perovskite and perovskite/Cu_2_O.

The energy band structure of the layers in a tandem solar cell is depicted in Fig. 1d, showing how the energy levels of the different materials align to allow electron flow. This begins with using FTO, a transparent conducting layer, to permit the passage of light into the solar cell. ZnO is used as the ETL to facilitate the flow of electrons between the Q-LEDs and the electrodes. The perovskite compound CsGeI_3_ is then used as the light-absorbing centre, converting incident light into electrical energy. Following the perovskite is Cu_2_O, acting as a hole-transporting layer and facilitating the movement of holes toward the electrodes. The bottom cell includes a heavily doped p+ silicon layer (Si(p+)) to improve charge-carrier collection at the back surface field. In the lower cell, light is absorbed in a second absorber layer; however, the Si(p) with heavy doping does not absorb light through the bulk since it is the primary absorber. Au was selected as a back contact due to its superior conductivity and stability, and because it is emitted from the n-type silicon layer above. Overall, this design clearly shows the need to adjust the energy band structures of the materials to minimize or eliminate energy barriers and maximize the free flow of charges, key factors in optimizing the design of a solar cell.

## Theoretical background

The simulation of our two-terminal CsGeI_3_/c-Si monolithic tandem solar cell relies on SCAPS-1D software, which numerically solves the fundamental equations governing charge transport, current generation, and key metrics like efficiency and fill factor^27,35^. SCAPS-1D employs finite-difference Newton-Raphson iteration to self-consistently couple Poisson’s equation with electron/hole continuity and drift-diffusion models. This captures steady-state carrier dynamics under AM1.5G illumination and applied bias, using realistic boundary conditions at selective contacts (ohmic ITO/ZnO; high-recombination Si(p^+^)/metal).

### Poisson equation

The distribution of the electrostatic potential inside the device is based on the Poisson equation, which relates the potential V(x) to the space charge density^36^. For a semiconductor device, such as a tandem solar cell, this is expressed as

1 $$\frac{d}{{dx}}\left( {\varepsilon \frac{{dV\left( x \right)}}{{dx}}} \right)= - q\left[ {p\left( x \right) - n\left( x \right)+{N_D} - {N_A}+{N_t}} \right]$$

where:

- $\:\epsilon\:$ is the permittivity of the material,
- * q* is the elementary charge,
- $\:p\left(x\right)$ and $\:n\left(x\right)$ are the position-dependent hole and electron concentrations, respectively,
- $\:{N}_{D}$ and $\:{N}_{A}$ are the donor and acceptor doping concentrations,
- $\:{N}_{t}$ represents the defect density contributing to trapped charges.

This equation ensures charge neutrality and dictates the electric field distribution across the layers, including the CsGeI_3_ perovskite top cell and the crystalline silicon (c-Si) bottom cell. The applied voltage and material interfaces, such as ZnO/CsGeI_3_ and Cu_2_O/Si, set the boundary conditions.

### Continuity equations

The transport of charge carriers (electrons and holes) is modelled using the continuity equations, which account for generation, recombination, and drift-diffusion processes^37^. For electrons and holes, these are given by:

2 $$\frac{{\partial n}}{{\partial t}}={G_n} - {R_n}+\frac{1}{q}\frac{{d{J_n}}}{{dx}}$$

3 $$\frac{{\partial p}}{{\partial t}}={G_p} - {R_p}+\frac{1}{q}\frac{{d{J_p}}}{{dx}}$$

where:

- $\:{G}_{n}$ and $\:{G}_{p}$ are the generation rates of electrons and holes due to photon absorption,
- $\:{R}_{n}$ and $\:{R}_{p}$ are the recombination rates,
- $\:{J}_{n}$ and $\:{J}_{p}$ are the electron and hole current densities, respectively.

In steady-state conditions (as simulated here), $\:\frac{\partial\:n}{\partial\:t}=0$ and $\:\frac{\partial\:p}{\partial\:t}=0$, simplifying the equations to balance generation, recombination, and current flow. The generation rate G is derived from the AM 1.5G solar spectrum and the absorption coefficient α of each layer, as^38^:

4 $$G\left( x \right)=\int \alpha \left( \lambda \right)\phi \left( \lambda \right){e^{ - \alpha \left( \lambda \right)x}}d\lambda$$

where $\:\phi\:\left(\lambda\:\right)$ is the photon flux at wavelength $\:\lambda\:$, and * x* is the depth within the absorber layer.

### Drift-diffusion equations

The current densities $\:{J}_{n}$ and $\:{J}_{p}$ are described by the drift-diffusion model, incorporating both electric field-driven drift and concentration gradient-driven diffusion^36^

5 $${J_n}=qn{\mu _n}E+q{D_n}\frac{{dn}}{{dx}}$$

6 $${J_p}=qp{\mu _p}E+q{D_p}\frac{{dp}}{{dx}}$$

where:

- $\:{\mu\:}_{n}$ and $\:{\mu\:}_{p}$ are the electron and hole mobilities,
- $\:E=-\frac{dV}{dx}$ is the electric field,
- $\:{D}_{n}=\frac{kT}{q}{\mu\:}_{n}$ and $\:{D}_{p}=\frac{kT}{q}{\mu\:}_{p}$ are the diffusion coefficients (via the Einstein relation),
- * k* is the Boltzmann constant, and * T* is the temperature (set at 300 K in this study).

These equations are critical for simulating the movement of charge carriers across the tandem structure, particularly at interfaces like CsGeI_3_/Cu_2_O and Si(p+)/Si(p), where band alignment affects carrier extraction efficiency.

### Current matching in tandem configuration

In a monolithic two-terminal tandem solar cell, the sub-cells are connected in series, requiring current matching to maximize efficiency. The total current density *J* is limited by the sub-cell with the lowest current^39^:

7 $$J={\mathrm{min}}\left( {{J_{top}},{J_{bottom}}} \right)$$

where, ${J_{top}}$ and ${J_{bottom}}$ are the current densities of the top (CsGeI_3_) and bottom (c-Si) cells, respectively. The short-circuit current density ${J_{sc}}$ is calculated as:

8 $${J_{sc}}=q\int\limits_{{{\lambda _1}}}^{{{\lambda _2}}} {EQE} \left( \lambda \right)\phi \left( \lambda \right)d\lambda$$

where $EQE\left( \lambda \right)$ is the external quantum efficiency optimized in this study to achieve ${J_{sc}}=23.69\,\frac{{{\mathrm{mA}}}}{{{\mathrm{c}}{{\mathrm{m}}^2}}}$ for the tandem device. The ${V_{oc}}$ is the sum of the sub-cell voltages:

9 $${V_{oc}}={V_{oc,\;top}}+{V_{oc,\;bottom}}$$

yielding $\:{V}_{oc}=1.89\:\mathrm{V}$ after optimization.

### Performance metrics

The power conversion efficiency (PCE) and fill factor (FF) are derived from the maximum power point (MPP) on the J–V curve^40^:

10 $$FF=\frac{{{J_{MPP}}.{V_{MPP}}}}{{{J_{sc}}.{V_{oc}}}}$$

11 $$\eta =\frac{{{P_{\hbox{max} }}}}{{{P_{in}}}}=\frac{{{J_{sc}}.{V_{oc}}.FF}}{{{P_{in}}}}$$

where $\:{P}_{in}=100\:\mathrm{m}\mathrm{W}/{\mathrm{c}\mathrm{m}}^{2}$ for AM 1.5G illumination. The simulations optimized these parameters to achieve FF = 82% and η = 37.23%, surpassing the Shockley-Queisser limit for single-junction cells.

## Results and discussion

### Energy band optimization of perovskite-based top and bottom cell

**Fig. 2:**
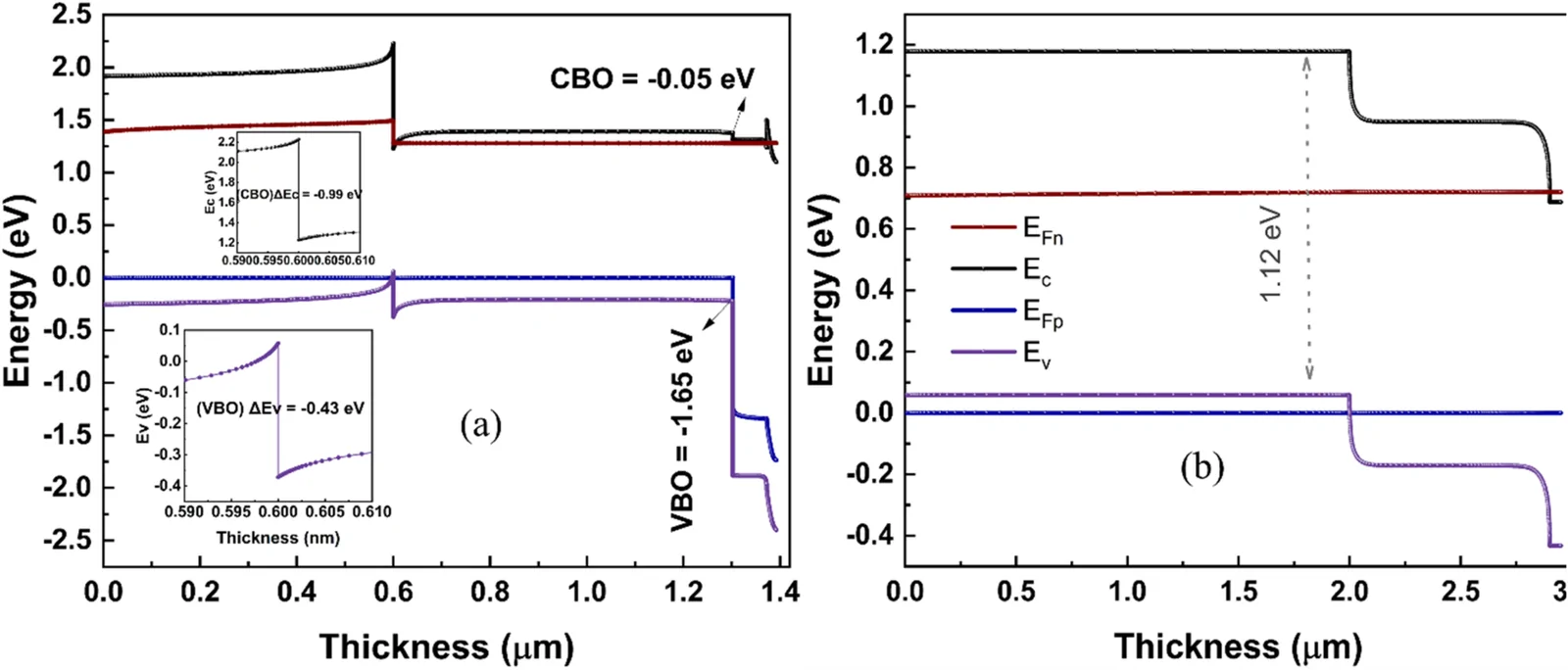
Energy level band diagram: (**a**) top cell and (**b**) bottom cell. The diagrams illustrate the conduction band (*E*_*c*_), valence band (*E*_*v*_), and Fermi levels (*E*_*Fn*_, *E*_*Fp*_), along with the conduction band offset (*CBO*) and valence band offset (*VBO*) values, highlighting key energy differences and material interfaces.

The simulated energy bands of the top and bottom cells is shown in Fig. 2. The energy band is a critical factor in analyzing efficiency since it affects how competently the charge carriers can be collected, transported, and generated. This work explores the EBD (energy band diagram) under A.M 1.5G sun illumination. This investigation aims to offer valuable insight into carrier dynamics occurring within the device and scrutinize its overall photovoltaic performance. Figure 2a,b revealed the functionality of EBD of the top and bottom cells obtained with short circuit illumination. The step-by-step alignment of energy levels varies depending on each material’s thickness. These figures provide an understanding of the quasi-fermi levels such as electron-Fermi energy level (E_fn_), hole-Fermi energy level (E_fp_), conduction energy level (E_c_), and n-type and p-type Fermi energy levels, respectively.

There is a clear indication of disparity in energy band alignment (Fig. 2a) at the ETL interface with perovskite; this discontinuity is known as conduction band offset (*CBO*). The negative offset (*CBO < 0*) creates a cliff-shaped barrier. It indicates the difference in the conduction band of two different layers. The significant difference in electron affinity between the layers could explain why this peak exists; electron affinity plays a vital role in device performance. The theoretical electron affinity of the perovskite layer is 3.52 eV, while after optimization, 4.2 eV provides good tunability to enhance device performance^19,20^. The electron affinity can be increased without changing the material’s bandgap by adjusting the doping concentration^41^. In Fig. 2a, a cliff barrier (CBO = −0.99 eV, VBO = −0.43 eV) is generated at the interface of HTL (Cu_2_O) and perovskite in both conduction and valence band energy levels. At the interface between two semiconductor layers, the conduction-band offset (CBO) and valence-band offset (VBO) strongly influence charge transport and recombination. A large negative band offset (cliff-type alignment) can enhance interfacial recombination by facilitating carrier back-transfer across the junction, which may reduce the open-circuit voltage (V_oc_). In contrast, a moderate positive offset (spike-type alignment) can act as a barrier that suppresses interfacial recombination, although excessively large spikes may hinder carrier transport. In the present simulation, the band alignment at the transport-layer/perovskite interfaces remains within an optimized range where carrier extraction is efficient while recombination losses are minimized. Despite the presence of slightly negative offsets, the optimized interface conditions lead to a high simulated V_oc_ of 1.89 V. Afterward, it will decrease the current density and efficiency of the device. Similarly, a well-aligned bottom cell EBD, shown in Fig. 2b, explains a significant upward shift of the valence and conduction band energy levels at the interface between layers. It will promote the hole, and the electron carries transfer from Si(p) to Si(p^+^). A good energy-band tunability between layers is required for improved device efficiency. These valuable observations illuminate the band alignment and electronic structure.

### The importance of absorber layer thickness on the performance of top and bottom cell

**Fig. 3:**
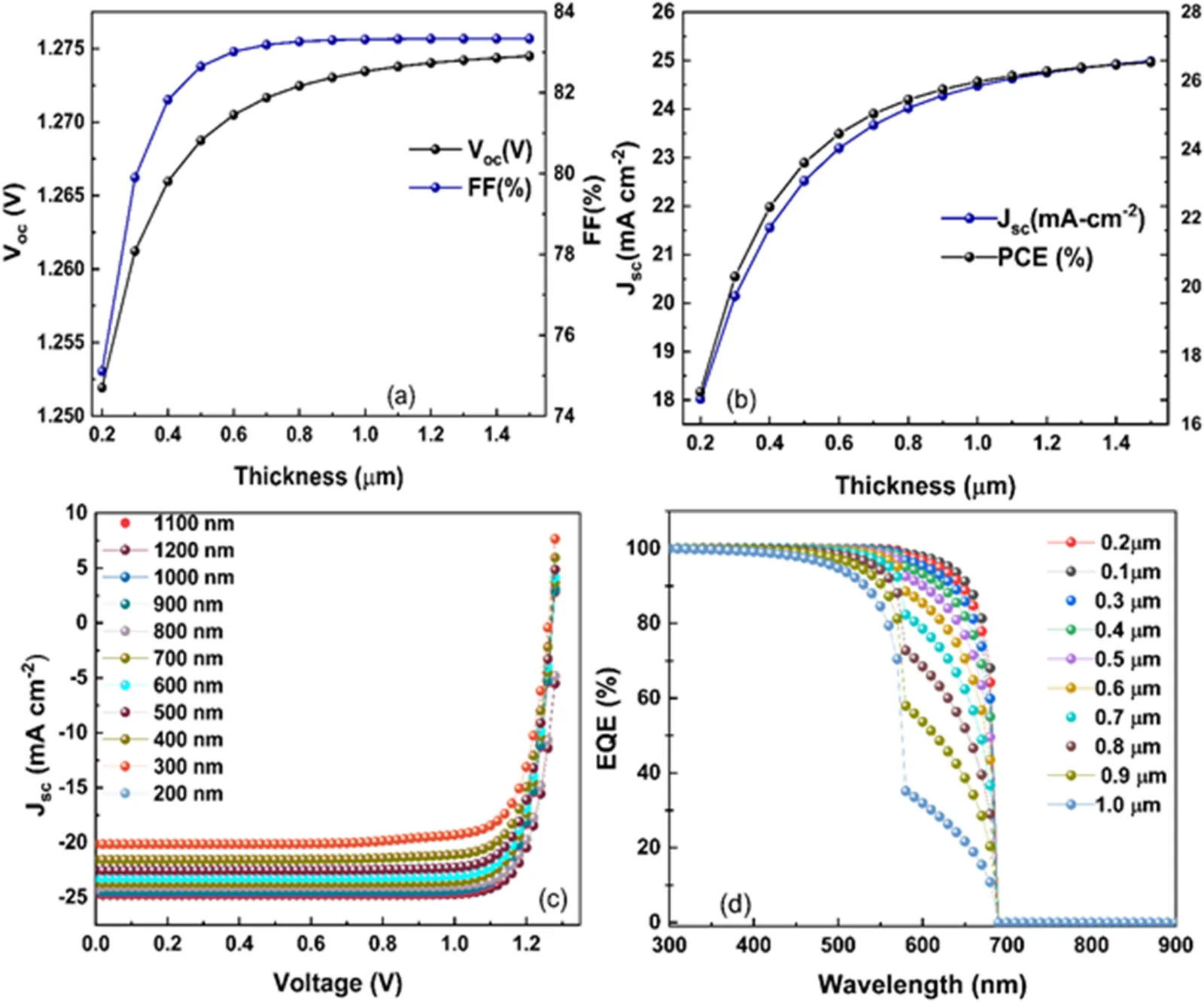
Simulation of top cell: (**a**) Variation in fill factor (FF) and open circuit voltage (V_oc_) with the thickness of the absorber CGI layer in the top cell. (**b**) Variation in J_SC_, PCE variation corresponds to the thickness of perovskite (**c**) J–V curve shows the variation with the thickness of the absorber layer under AM 1.5G illumination. (**d**) repsresent the EQE dependence on the thickness of the absorber layer of the CGI single junction top cell.

**Fig. 4:**
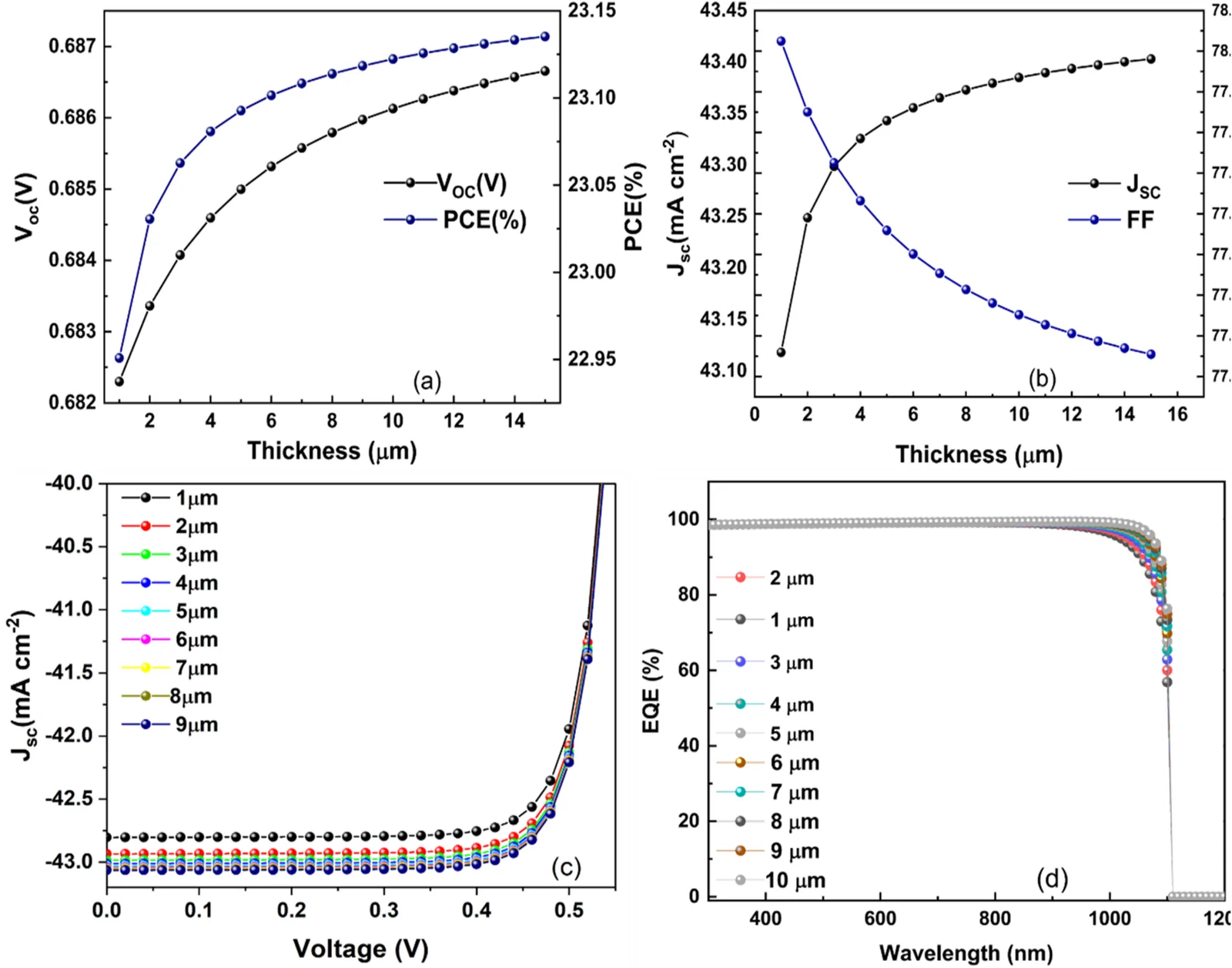
Simulation of bottom cell: (**a**) Variation in *V*_*OC*_ and *PCE* corresponds to the thickness of the absorber CGI layer of the top cell. (**b**) Variation of *J*_*SC*_ and *FF* with thickness of Perovskite (**c**) *J-V* curve with thickness under AM 1.5G illumination. (**d**) *EQE* deviation with the thickness of the absorber layer CGI.

This subsection provides a detailed examination of the top and bottom cells before evaluating the results of the two-terminal solar cells. This section examines the impact of varying the thickness of the absorber layer in both subcells of the device. Figures 3a–d and 4a–d demonstrate significant alterations in both cells’ performance parameters and efficiency. This study presents the results of a single-junction top cell (Fig. 3) and a bottom cell (Fig. 4). We examined the performance of all devices regarding the parameters *J*_*SC*_, *V*_*OC*_, *FF*, and *PCE* by varying the thickness of the absorber layer. Only the thickness of the absorber layer is modified, while all other values in Tables 1 and 2 remain constant throughout the simulation.

**Table 1:** All material parameters which employed in numerical simulation work^42,43^.

Parameter	FTO	Cu_2_O	CsGeI_3_	ZnO	Si (*P*+)	Si (*P*)	Si (*n*)
Thickness(µm) – 2T	0.020	0.600	Variable	0.070	Variable	Variable	Variable
Bandgap (eV) E_g_	3.5	2.17	1.6	3.5	1.12	1.12	1.12
Electron affinity (eV)	4.0	3.2	3.52	4.26	4.05	4.05	4.05
Dielectric permittivity ∈r	9.0	7.5	18.0	9.0	11.9	11.9	11.9
Electron mobility (cm^2^ /V. s)	20	20	20	100	1400	1400	1400
Hole mobility (cm^2^ /V. s)	10	80	20	25	450	450	450
Donor concentration, Nd (cm^−3^)	1x$\:{10}^{18}$	0E + 0	1x$\:{10}^{16}$	1x$\:{10}^{18}$	0.0E + 0	0.0E + 0	1x$\:{10}^{20}$
Acceptor concentration, Na (cm^−3^)	0.0E + 0	1 × 10^14^	0.0E + 0	0.0E + 0	1 × 10^20^	1.0x$\:{10}^{16}$ 1 × 10^16^	0.0E + 0
CB conduction band density of states Nc (cm^−3^)	1 × 10^15^	2.2 × 10^18^	1 × 10^18^	4 × 10^18^	2.819 × 10^19^	2.819 × 10^19^	2.819 × 10^19^
VB valence band density of states N_c_ (cm^−3^)	2.2 × 10^18^	1.8 × 10^19^	1 × 10^19^	1 × 10^19^	1.04 × 10^19^	1.04 × 10^19^	1.04 × 10^19^
Total defect density N_t_ (cm^−3^)	0.0E + 0	1E14	1E15	1E14	1E15	1E15	1E15

**Table 2:** All defects parameter of materials^41^.

Parameters	IDL1 CsGeI_3_/ZnO	IDL2 Cu_2_O/CsGeI_3_	IDL3 c-Si(*p*)/c-Si(*n*)	IDL4 c-Si(*p*+)/c-Si(*n*)	Bulk CsGeI_3_
Defect type	Neutral	Neutral	Neutral	Neutral	Neutral
Energetic distribution	Single	Single	Single	Single	Single
Capture cross-section of holes (cm^2^)	1 × 10^15^	1 × 10^15^	1 × 10^15^	1 × 10^19^	1 × 10^18^
Capture cross-section of electron (cm^2^)	1 × 10^19^	1 × 10^19^	1 × 10^19^	1 × 10^18^	1 × 10^18^
Energy level reference	Above highest E_v_	Above highest E_v_	Above highest E_v_	Above highest E_v_	Above highest E_v_
Energy level (eV)	0.600	0.600	0.600	0.600	0.600

The CGI layer’s thickness variation is 0.2–1.5 μm. The results indicate that, starting at 0.3 μm, all four performance parameters of the top cell increase until the thickness reaches 0.5 μm. As the thickness increases a large number of absorption photons will then collect in the active layer, generating more charge carriers. The increment in *J*_*SC*_ can be attributed to an optimized band gap and a large number of valence and conduction band levels in perovskite materials. The efficiency gradually increases and reaches a constant value of ~ 25%. Exceeding 0.5 μm (a critical thickness), all parameters enter a saturation state due to the formation of defects in the bulk or excessive recombination of electron-hole pairs. Similarly, we simulate a single-junction bottom cell and analyze *J*_*SC*_, *V*_*OC*_, *FF*, and *PCE* as a function of thickness, as shown in Fig. 4a,b. The values of *J*_*SC*_, *V*_*OC*_, and *PCE* increased until the thickness of the absorber layer reached close to the diffusion length of charge carriers. Throughout this period, *FF* declines with increasing thickness; however, the relative change is not significant. It is observed that the *PCE* of the silicon bottom cell gradually increased and reached a plateau of around 23%. CGI perovskite simulation evidence matched results reported in many published articles^44,45^. It demonstrates that the perovskite cell’s efficiency reached a plateau after a thickness of 1 μm. In contrast, the bottom cell shows the exact efficiency behavior at a thickness of 10 μm. This happens because the silicon (E_g_ = 1.1 eV) absorber layer needs a greater thickness to absorb many photons than the perovskite layer. The nature of the current density and voltage of both cells changes as the thickness of the absorber layer is varied. In this work, we demonstrated the *J-V* behavior of single junction top and bottom cells in Figs. 3 and 4c The short circuit current density of the top cell, shown in Fig. 3c, expanded from − 24.3 to −20.14 mA-cm^−2^ while *V*_*OC*_ in the range of 0–1.3 volts due to making a change in the thickness of the CGI layer from 0.2 to 1.1 μm in the top cell. Similarly shown in Fig. 4c, when the thickness of the active layer is modified from 1 μm to 9 μm, then the variation range of J_SC_ is − 42.80 mA-cm^−2^ to − 43.05 mA-cm^−2^, and V_OC_ is 0–0.6 V. As we explained above, silicon absorbs more radiation because of its small bandgap. The wavelength range of absorption photons can be understood from the external quantum efficiency curves in Figs. 3d and 4d. The bottom cell spans of broad wavelength range from 200 nm to 1100 nm (due of its low bandgap of 1.1 eV), which is associated with the higher J_SC_ value. The bottom cell’s *EQE* report suggests that the device provides a sound output of around 10 μm. Through *EQE*, we can get an idea of the successful conversion rate of absorbed photons into electric current. Figure 4d shows *EQE* shift with a change in the absorber layer’s thickness from 0.1 μm to 1.1 μm. *EQE* decreases rapidly as the thickness exceeds an optimal value of 0.5 μm because thicker layers generate more charge carriers, leading to unwanted recombination. It begins to block the cell’s light-trapping mechanism. The innate changes shown in the above *J-V* plots clearly indicate that both single-junction devices exhibit a significant current-matching gap.

### Impact of defect density and defect energy level on single junction top cell

**Fig. 5:**
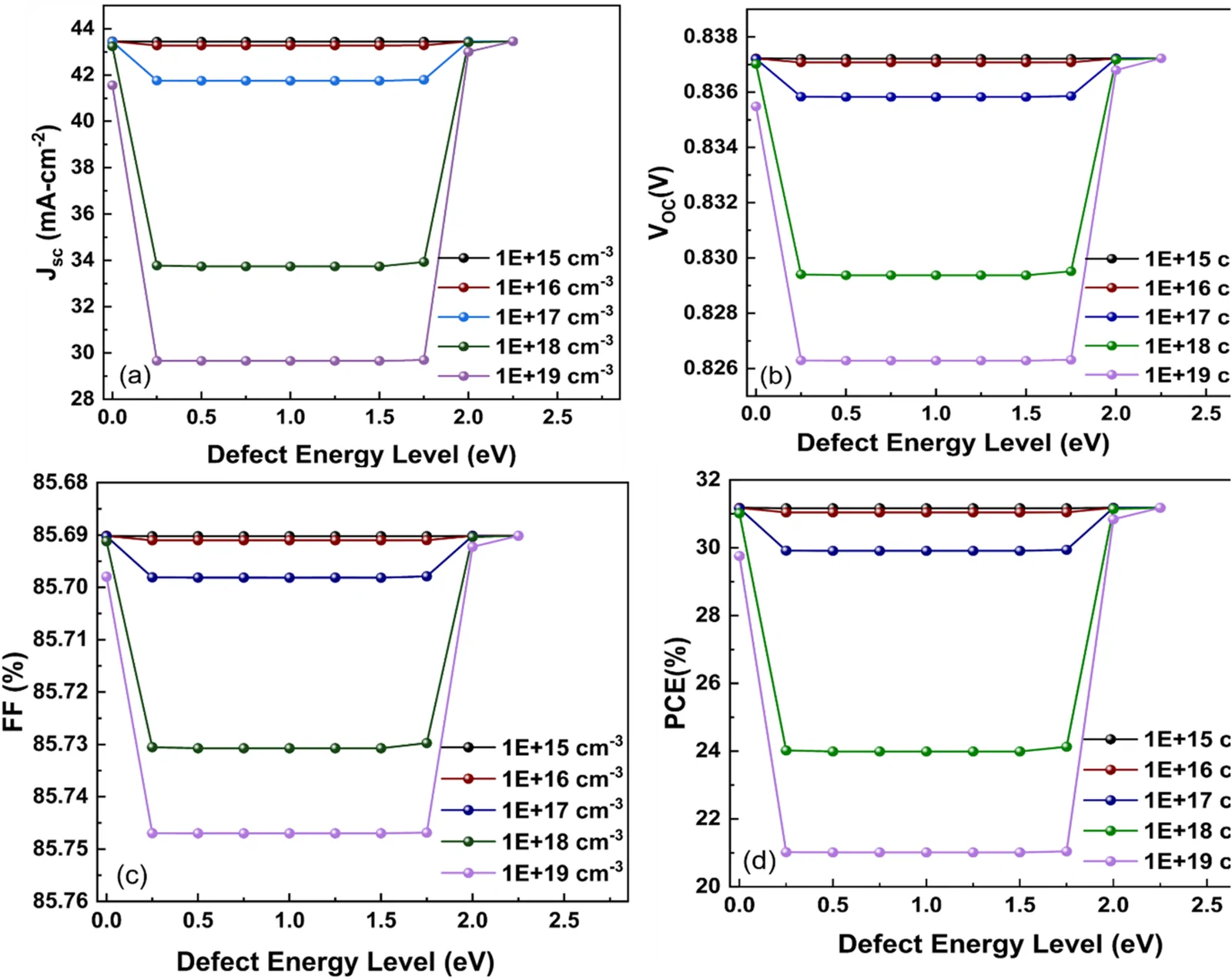
Device performance under various defect density concentrations: Device functionality in the CsGeI_3_ absorber layer at varying defect energy levels, showing (**a**) short-circuit current density (J_SC_), (**b**) open-circuit voltage (V_OC_), (**c**) fill factor (FF), and (**d**) power conversion efficiency (PCE). Each plot examines performance metrics across different defect density concentrations.

The findings suggest that defect states play a critical role in shaping the device’s performance. Generally, these defect states are found in the material bandgap, which results in the unfavorable trapping or scattering of mobile charge carriers, fundamentally causing the reduction in device parameters^46,47^. Figure 5 shows the change in device functionality by using different concentrations of defect density at defect energy levels. The effect of deep-defect density concentration from 10^15^ to 10^19^ cm^−3^ is observed. When the deep-defect density rises to 10^17^ cm^−3^, a notable performance parameter decline is observed by ~ 45%. The impact of increasing concentration of defect density on the occurrence of charge recombination in the top cell is evident^48^.

From Fig. 5, it can be observed that the short-circuit current density is highest and most stable for defect density concentrations of 1 × 10^15^ cm^−3^, 1 × 10^16^ cm^−3^, and 1 × 10^17^ cm^−3^, with a slight drop at 1 × 10^18^ cm^−3^ and a significant drop at 1 × 10^19^ cm^−3^. The V_oc_ is highest and stable for defect density concentrations up to 1 × 10^18^ cm^−3^, with a significant drop at 1 × 10^19^ cm^−3^. The fill factor remains high and stable for defect density concentrations up to 1 × 10^18^ cm^−3^, with a decline at 1 × 10^19^ cm^−3^. The PCE is highest and stable for defect density concentrations up to 1 × 10^18^ cm^−3^, with a significant drop at 1 × 10^19^ cm^−3^. Based on these observations, the optimum defect density concentration appears to be around 1 × 10^15^ cm^−3^ to 1 × 10^17^ cm^−3^. This range provides the highest and most stable performance across all metrics, including *J*_*SC*_, *V*_*OC*_, *FF*, and *PCE*, without the performance degradation seen at higher defect density concentrations.

### Effect of temperature on tandem solar cell and subcells

**Fig. 6:**
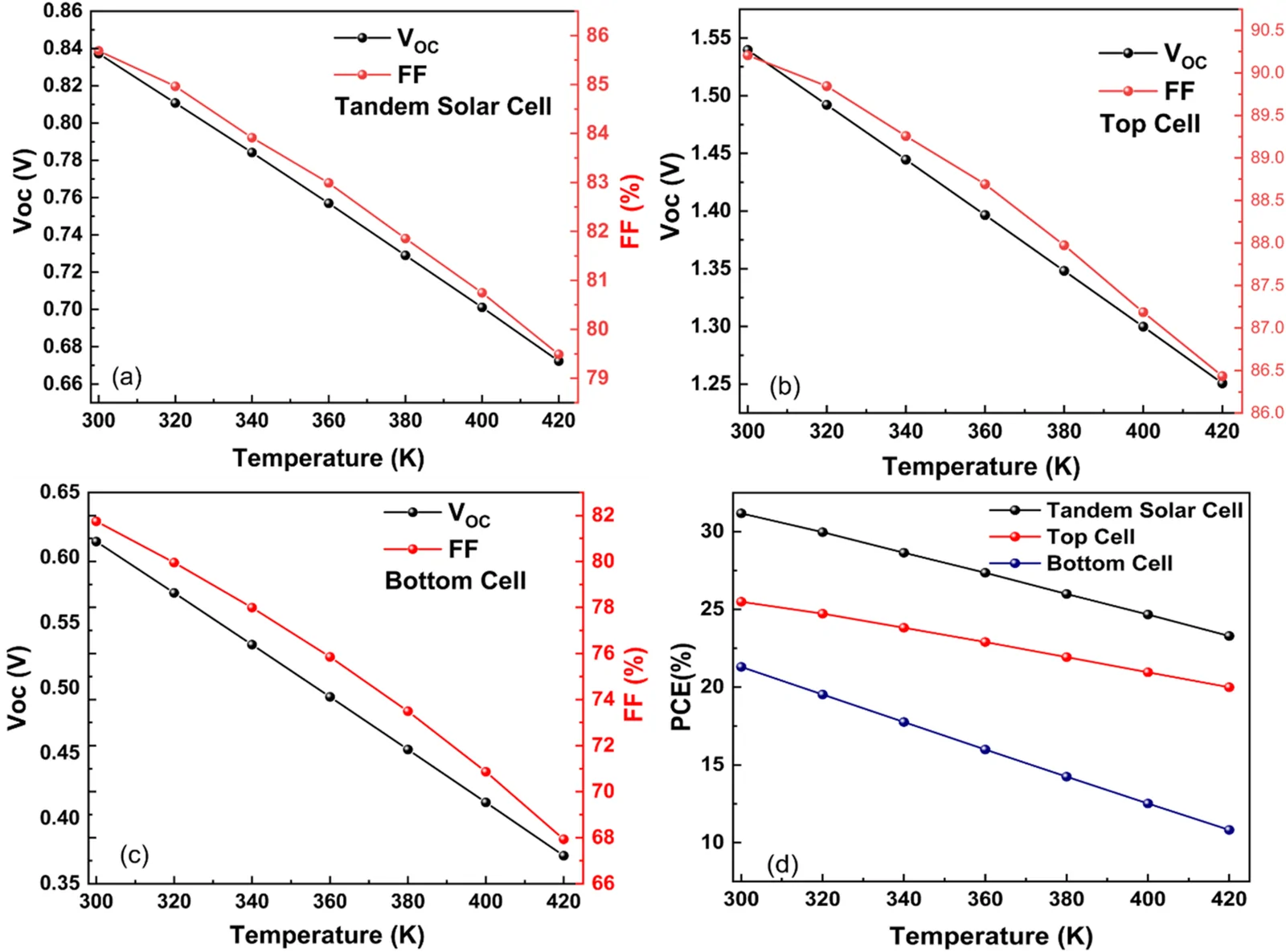
Temperature effects on solar cell performance: (**a**) open-circuit voltage (*V*_*OC*_) and fill factor (*FF)* in the tandem cell, (**b**) *V*_*OC*_ and *FF* in the top cell, (**c**) *V*_*OC*_ and *FF* in the bottom cell, and (**d**) power conversion efficiency (*PCE*) decrease in all cells. The graphs illustrate the decline in performance metrics as temperature increases from 300 to 420 K.

Figure 6 illustrates the effect of temperature on the TSC, the Top Cell’s absorber layer (CsGeI_3_), and the bottom cell’s absorber layer, the Si(P) layer. Table 3 presents the corresponding decrease in the performance parameters of all devices. To observe the characteristics, the temperature varies from 300 to 420 K, keeping all the parameters constant.

The increase in temperature from 300 to 420 K had an influence on the performance of the Tandem Solar Cell, the Top Cell absorber layer (CsGeI_3_), and the Bottom Cell absorber layer (Si(P)). In addition to the improvements in current density in all layers with the increase in temperature; the overall efficiency of each device decreased. A reduction was also observed in the FF and in the V_oc_ in all three devices^47,49^. This degradation in device performance with increasing temperature can be explained by several physical mechanisms. As temperature increases, bandgap narrowing occurs, which leads to a reduction in V_oc_. Additionally, higher temperatures increase the intrinsic carrier concentration, which enhances recombination losses within the device. Furthermore, elevated temperatures strengthen phonon scattering, which reduces carrier mobility and contributes to the degradation of the FF and overall device efficiency. Although a slight increase in current density can occur due to enhanced carrier generation at higher temperatures, the overall effect results in reduced device performance. Therefore, the temperature of operation is another parameter that affects the performance of the device. The results of Fig. 6d illustrate the highest PCE for this tandem solar cell. The tandem solar cell consists of a perovskite top cell and a silicon bottom cell. The integration of these two cells allows for improved collection of current through the absorption of different parts of the solar spectrum. Therefore, it is important to maintain a stable temperature range to achieve the maximum possible efficiency from tandem solar cells.

In addition to the previously mentioned effects of increasing temperature on the tandem cell, a degradation of V_oc_ and FF was observed for the tandem cell as the temperature increased above 300 K. These values decrease further as the temperature continues to rise toward the highest temperature tested (420 K). V_oc_ and FF exhibit their highest values at 300 K and gradually decrease with increasing temperature, primarily due to enhanced carrier recombination and bandgap narrowing at elevated temperatures. The Voc and FF of the bottom cell exhibit a similar behavior pattern to the tandem cell, although the reduction in performance occurs more rapidly compared with the tandem device. The performance of the bottom cell was therefore found to be better at lower temperatures such as 300 K. In all cases, a decrease in *PCE* occurred as the temperature increased. The highest efficiencies were achieved at 300 K. Therefore, the optimal temperature for operating these solar cells appears to be at or near 300 K. All of the performance parameters of the cells demonstrate their greatest *V*_*OC*_, *FF* and *PCE* at this temperature, demonstrating the best possible performance of the cells. Beyond this temperature, a significant decline in all of the performance parameters was observed.

**Table 3:** Variation in performance parameters from 300 to 400 K, highlighting the impact of temperature on each device independently.

Devices	FF (%) (decrease by)	PCE (%) (decrease by)	V_OC_ (V) (decrease by)
Tandem cell	6.2	7.8	0.16
Top cell	3.7	5.5	0.28
Bottom cell	13.8	10.5	0.24

### Perovskite-silicon tandem solar cell

This section thoroughly explains the simulations conducted on TSC, which achieve exceptionally high efficiency by integrating top and bottom cells. Numerous studies have shown that incorporating silicon as the bottom cell in a tandem device enhances performance outcomes^50^. Currently, no empirical data is supporting the use of tandem solar cells that combine Copper Gallium Indium Selenide (CGI) with silicon solar cells. However, numerous empirical and theoretical investigations using SCAPS or Density Functional Theory (DFT), have highlighted the impressive efficiency of single-junction CGI solar cells^42^. Conducting experimental research on CGI-silicon tandem solar cells to analyze device performance would be highly intriguing. In a monolithic tandem device, the top and bottom cells are two diodes connected in series. In this setup, the current flowing through both diode cells must be identical, while the total V_oc_ equals the sum of the individual V_oc_ of each subcell^51^.

**Fig. 7:**
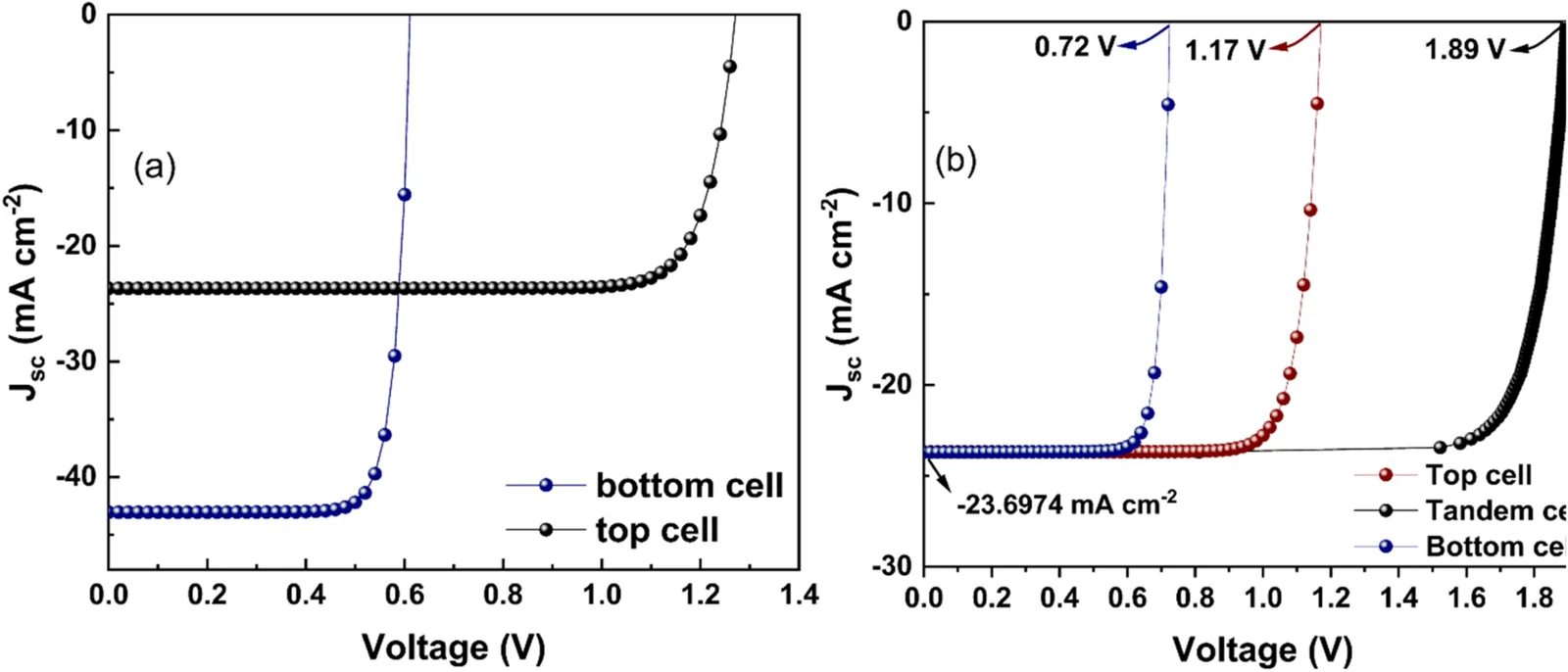
Current-Voltage (J-V) characteristics of top, bottom and tandem cells (**a**) Highlights a noticeable difference in the *J-V* curves between the top and bottom cell configurations. This discrepancy prevents SCAPS from generating a tandem *J-V* diagram for the affected region. (**b**) Shows distinct current-voltage (*J-V*) curves for the upper and lower cells, pinpointing the optimal thickness needed to achieve current matching using a tandem script file technique.

The data shown in Fig. 7a, illustrate the substantial differences in the current densities of the upper and lower cells of the tandem structure. These are 23.60 mA/cm^2^ (upper cell represented by blue), and 43.04 mA/cm^2^ (lower cell represented by red). Thus, there is a significant difference in the current densities of the upper and lower cells. A way to achieve smooth integration of both cells and equalize the currents at their junctions could be to adjust the thicknesses of the active layers of the two cells. In addition, these adjustments should account for the absorption spectras of each of the cells. In order to test the performance of the upper cell of the monolithic tandem solar cell, it was exposed to ~1sun, AM 1.5 G spectrum light illumination. Tandem device fabrication requires sequential deposition, with the bottom c-Si cell performance assessed under a filtered AM1.5G spectrum that accounts for photon absorption by the top CsGeI_3_ layer. This spectral filtering reduces the silicon sub-cell’s short-circuit current density from 43 to ~ 23.70 mA/cm^2^, as modeled by decreased photon flux transmission. Current matching in the monolithic two-terminal design is achieved by optimizing layer thicknesses or adjusting illumination conditions. The top cell receives full AM1.5 G illumination, while the bottom cell operates on the transmitted spectrum filtered through optical losses from the upper perovskite. The tandem configuration pushes the upper cell to absorb high-band-gap light, and the incident light that has passed through the upper cell is absorbed by the lower cell^51^. The current density of the lower cell can then be adjusted and fine-tuned by adjusting the thickness of the absorber layer in the lowest sub-cell.

The final analysis of the optimized thickness configuration is shown in Fig. 7b. It shows the following thicknesses: 0.702 μm for the CsGeI_3_ (active layer), 0.07 μm for the ZnO (HTL), and 0.6 μm for the Cu_2_O (HTL) in the upper cell; and 0.702 μm for the S (p) absorber layer, 0.06 μm for the Si (p+) layer, and 0.05 μm for the Si (n) layer in the lower cell. This configuration results in a *J*_*SC*_ of 23.695 mA/cm^2^. This configuration gives a cumulative voltage of 1.89 V in the tandem device. The thickness of the absorber layer in the bottom cell plays a crucial role in achieving current matching in the tandem configuration. Under optimized conditions, the simulated tandem device exhibits a power conversion efficiency (PCE) of 37.23% with a fill factor (FF) of 82%, surpassing the performance of the corresponding single-junction devices. This efficiency represents the theoretical maximum predicted by SCAPS simulations, assuming some ideal condition with perfect stability of Ge^2+^ in CGI. Experimentally, achieving certified efficiencies exceeding 25% is considered feasible through effective surface passivation and processing under inert conditions. Further support the potential pathway from simulation to practical tandem device fabrication. To validate the reliability of the simulation model, the obtained photovoltaic parameters were compared with previously reported SCAPS simulation studies of similar tandem solar cell configurations. The comparison shows that the simulated values fall within the expected range of reported results, confirming the consistency of the model. Recent studies have demonstrated the potential of monolithic tandem solar cells designed to optimize their performance, which is supported by the literature and the results from this study, as shown in Table 4.

**Table 4:** Comparison of TSC SCAPS simulation results as reported in published research.

Top cell	Bottom cell	V_OC_ (V)	J_SC_ (mA cm^−2^)	FF (%)	PCE (%)	Reference
Cs_2_AgBi_0.75_Sb_0.25_Br_6_	FACsPb_0_._5_Sn_0_._5_I_3_	1.83	14.90	63.57	17.35	^52^
MAPbI_3_	c-Si	1.92	18.11	79.45	27.66	^53^
MAGeI_3_	FAMASnGeI_3_	0.94	29.36	83.20	23.10	^54^
MAGeI_3_	c-Si	2.06	16.20	85.90	28.71	^38^
CsSn_0.5_Ge_0.5_I_3_	c-Si	1.72	20.02	83.74	28.53	^56^
CsGeI_3_	c-Si	1.89	23.69	82.00	37.23	This work

The comparison of Tandem Solar Cell (TSC) SCAPS simulation results from various published studies demonstrates significant advancements in achieving high *PCE* with different material combinations (Table 4). The combined performance data in Table 4 suggests that selecting both top and bottom cells is key to achieving good overall device performance. Among the combinations studied, this work demonstrated the highest *PCE* of 37.236% for the CsGeI_3_/c-Si tandem configuration, showing superior efficiency. This highlights the potential for maximizing the benefits by optimizing contacts, materials, and architecture in tandem cells. These results require further investigation through empirical work and simulations, as new material combinations may lead to even higher efficiencies.

## Conclusion

The findings from this modeling and simulation study of monolithic two-terminal tandem solar cells using CsGeI_3_-based top cells on c-Si bottom cells are providing climate-stable solutions to meet growing needs for sustainable energy. The results indicate that the new tandem solar cells show greater efficiencies than those of traditional lead-based solar cells. The top cell demonstrated an efficiency of 25.75% at a *V*_*OC*_ of 1.27 V, *FF* of 83.33%, and a *J*_*SC*_ of 24.27 mA/cm^2^ (FTO/Cu_2_O/(1.6 eV) perovskite/ZnO), while the bottom cell showed a *V*_*OC*_ of 0.64 V, *JSC* of 43.17 mA/cm^2^, FF of 81.15%, and PCE of 22.53%. The tandem solar cell demonstrated a *V*_*OC*_ of 1.89 V, *J*_*SC*_ of 23.69 mA/cm^2^, *FF* of 82.00%, and *PCE* of 37.23%. In addition, we investigated the influence of defect densities and doping concentrations as a way to enhance the performance of the device. We found that this two-junction device will be able to demonstrate a significant increase in its *PCE* to 37.23%, which is an all-time best. Our study will address the issues related to the experimental setup and provide the basis for further studies on high-performance tandem solar cells. The simulations presented here could be used to improve the development of tandem solar cells for the purpose of increasing the efficiency of solar energy conversions and greatly contribute to the use of renewable energy.

## Data Availability

The datasets generated and/or analyzed during the current study are not publicly available due to further research going on, but are available from the corresponding author on reasonable request.
